# Supplementary material for: Test-Negative Designs with Multiple Testing Sources
Source: Res Sq. 2025 Apr 29:rs.3.rs-6548614. Preprint. [Version 1] doi: 10.21203/rs.3.rs-6548614/v1 (PMC12060981; doi:10.21203/rs.3.rs-6548614/v1)
Supplement: Supplement 1 [file NIHPPrs6548614v1-supplement-1.pdf]

APPENDIX

ALGORITHM

Notably, if we independently solve the two distinct loss functions presented in sections 2.1 and 2.2, respectively, we arrive at a consistent estimator for the parameters (p_0', q_0', λ'). This consistent estimator can be selected as the initialization point for our gradient descent algorithm. This strategy leads to the first phase of the algorithm, which is concerned with initialization, as detailed in the subsequent discussion.:

Step 1, Initialization: We first solve $\hat{p}_0^{(0)}, \hat{p}_1^{(0)}, \hat{q}_0^{(0)}, \hat{q}_1^{(0)}$ by maximizing likelihoods (2.1) and (2.2), respectively. We then initialize the re-parameterized parameters at the zeroth (initial) step $(\hat{p}_0'^{(0)}, \hat{q}_0'^{(0)}, \hat{\lambda}'^{(0)})$. In specific, we first let $\hat{p}_0'^{(0)} = \log(1 - 1/\hat{p}_0^{(0)}), \hat{q}_0'^{(0)} = \log(1 - 1/\hat{q}_0^{(0)})$ and initialize the estimator of $\hat{\lambda}'^{(0)}$ by an log- weighted average of $\hat{\lambda}_1, \hat{\lambda}_2 : \log(w_1\hat{\lambda}_1 + w_2\hat{\lambda}_2)$, where $\hat{\lambda}_1 = (\frac{\hat{p}_1^{(0)}}{1-\hat{p}_1^{(0)}})/(\frac{\hat{p}_0^{(0)}}{1-\hat{p}_0^{(0)}})$ and $\hat{\lambda}_2 = \hat{q}_1^{(0)}/\hat{q}_0^{(0)}$. The weights w_1, w_2 with $w_1 + w_2 = 1$ are pre-determined. It can be chosen equally or proportional to the standard deviation of $\hat{\lambda}_1$ and $\hat{\lambda}_2$, respectively.

Following the initialization phase, we progress to the subsequent stage, which entails the execution of the gradient descent algorithm. The specifics of this step are elucidated in the following description.

Step 2, Run Gradient Descent: Choose stepsize η and run gradient descent on the parameters p_0', q_0', λ'. The updates are presented as follows:

$$(\hat{p}_0'^{(t+1)}, \hat{q}_0'^{(t+1)}, \hat{\lambda}'^{(t+1)}) = (\hat{p}_0'^{(t)}, \hat{q}_0'^{(t)}, \hat{\lambda}'^{(t)}) - \eta\left(\nabla_{p_0'}\mathcal{L}(\theta^{(t)}), \nabla_{q_0'}\mathcal{L}(\theta^{(t)}), \nabla_{\lambda'}\mathcal{L}(\theta^{(t)})\right)$$

where $\theta^{(t)} = (\hat{p}_0'^{(t)}, \hat{q}_0'^{(t)}, \hat{\lambda}'^{(t)})$. The gradient is given as follows:

$$\begin{aligned}
\nabla_{p_0'}\mathcal{L}(\theta) &= -\left[(C' + D') - (B' + D')\frac{\exp(p_0')}{1 + \exp(p_0')} - (A' + C')\frac{\exp(p_0')}{\exp(\lambda') + \exp(p_0')}\right] \\
\nabla_{q_0'}\mathcal{L}(\theta) &= -\left[(A^* + B^* + C^* + D^*)\frac{\exp(q_0')}{1 + \exp(q_0')} - D^* - \frac{B^*\exp(q_0')}{1 + \exp(q_0') - \exp(\lambda')}\right] \\
\nabla_{\lambda'}\mathcal{L}(\theta) &= -\left[A' - \frac{(A' + C')\exp(\lambda')}{\exp(\lambda') + \exp(p_0')} + A^* - \frac{B^*\exp(\lambda')}{1 + \exp(q_0') - \exp(\lambda')}\right],
\end{aligned}$$

where $\theta = (p_0', q_0', \lambda')$.

Step 3, Stopping Criterion: Upon reaching a point in the iterative process denoted as $\theta^{(t)}$, we terminate the algorithm when the infinity norm of the gradient at that particular iterate falls below a predetermined threshold value, denoted as τ.

In the simulation experiments, we choose a step size of $\eta = 0.01/n$, and a stopping threshold of $\tau = 10^{-5}$ is used.

THEORETICAL GUARANTEES

In the previous simulation example, we observed that our estimator outperformed weighted alternatives in terms of both finite sample bias and standard deviation.

By investigating the theoretical guarantee of the asymptotic variance, we aim to provide a deeper understanding of the statistical properties and performance limits of our proposed estimator. This analysis will shed light on the precision and reliability of our estimator as sample sizes grow and allow for valuable insights into its practical applications.

.1 Uncertainty Quantification and Cramer Rao Lower bound

Since our joint estimator $\hat{\theta}$ minimizes the negative log-likelihood (2.3), according to Taylor Expansion, we know

$$0 = \nabla\mathcal{L}(\hat{\theta}) = \nabla\mathcal{L}(\theta^*) + \nabla^2\mathcal{L}(\theta^*)(\hat{\theta} - \theta^*) + \text{ higher order terms}$$

where $\theta^* = (p_0, q_0, \lambda)$. By direct calculation, we obtain

$$\hat{\theta} - \theta^* = \frac{-\nabla\mathcal{L}(\theta^*)}{\nabla^2\mathcal{L}(\theta^*)} + o_p(1).$$

All involved distributions are binary distributions with bounded support. Moreover, the density function is also continuous to $\theta = (p_0, q_0, \lambda)$. Therefore, the regularity condition holds and $\mathrm{Var}(\nabla\ell(\theta^*)) = \mathbb{E}[\nabla^2\ell(\theta^*)]$ (since we are studying the negative log-likelihood). Therefore, we have the following theorem for the asymptotic distribution of $\hat{\theta}$.

Theorem 1. *We have $\hat{\theta} - \theta^* \to N(0, I^{-1}(\theta^*))$ where $I(\theta^*) = \mathbb{E}[\nabla^2\ell(\theta^*)]$ is the Fisher information matrix evaluated under θ^*.*

Based on the presented theorem, we can deduce that our joint estimator has the capability to achieve the minimum variance among all consistent estimators. This finding provides a theoretical validation for our previous observation that our estimator exhibits superior efficiency in terms of standard deviation. It confirms that our estimator possesses favorable statistical properties, reinforcing its potential as an optimal choice for parameter estimation in the given context.

Next, we use some simulations to validate the asymptotic variance in the following section.

SIMULATION VALIDATION

In this section, we validate the asymptotic variance of $\hat{\lambda}_c$ presented in Theorem 1. In this simulation, we employ the same settings as outlined in Section 3. We repeat the experiments 1000 times and record the normalized estimators, which are obtained by dividing the estimators by the corresponding variances presented in Theorem 1, for each replication. The resulting normalized estimators are then used to construct histograms, which are presented below.

The presented figures consist of two panels in Figures 1 and 2 , corresponding to experiments conducted with sample sizes of $n = 500$ and $n = 1000$, respectively. Each figure contains five rows, representing the results obtained for different true parameter values $\lambda = 1, 0.9, 0.8, 0.7, 0.6$.

These histograms provide an empirical visualization of the distribution of the normalized estimators, offering insights into the accuracy and precision of our proposed methodology under different true parameter settings and sample sizes.

FIGURE 1 Asymptotic distribution of normalized $\hat{\lambda}_c - \lambda$ under different cases. (a): $\lambda = 1.0, n = 500$; (b): $\lambda = 1.0, n = 1000$; (c): $\lambda = 0.9, n = 500$; (b): $\lambda = 0.9, n = 1000$;

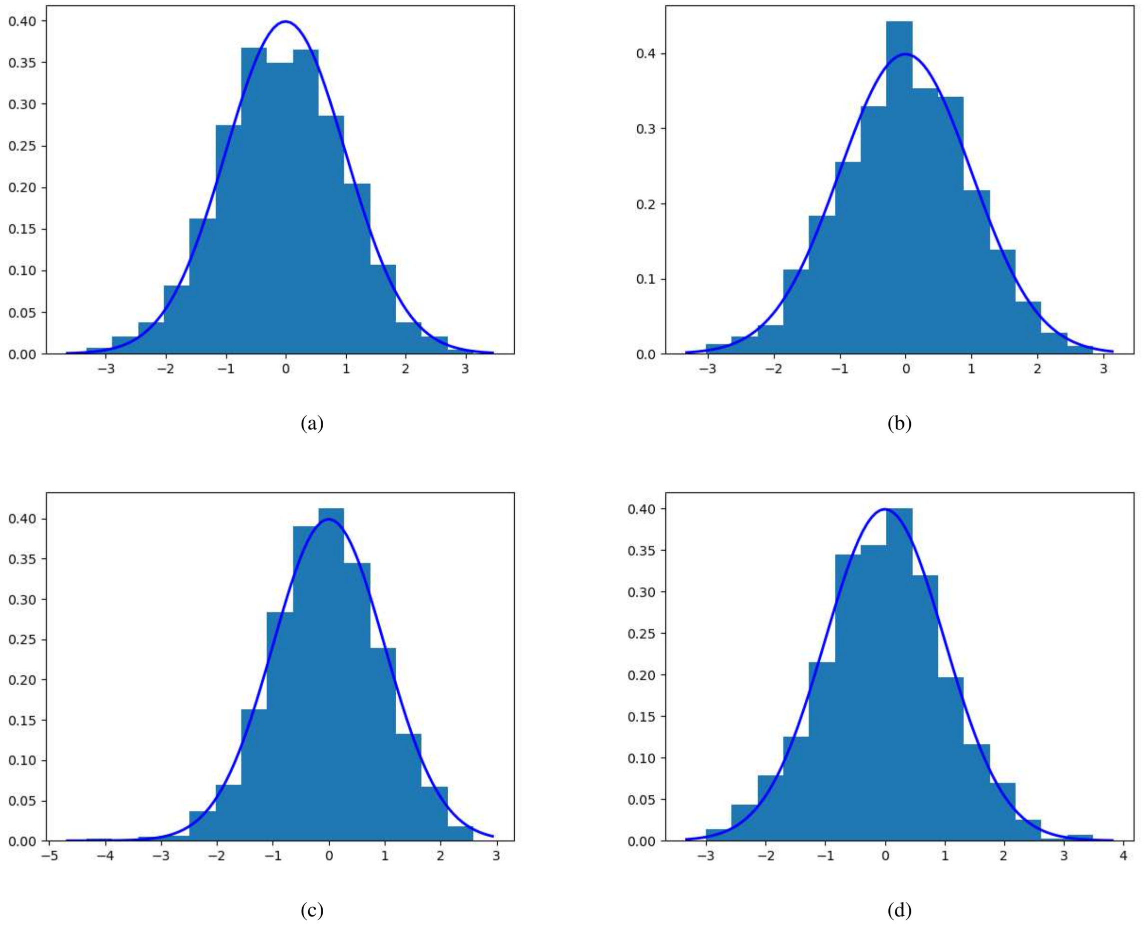

FIGURE 2 Asymptotic distribution of normalized $\hat{\lambda}_c - \lambda$ under different cases. (e): $\lambda = 0.8, n = 500$; (f): $\lambda = 0.8, n = 1000$; (g): $\lambda = 0.7, n = 500$; (h): $\lambda = 0.7, n = 1000$; (i): $\lambda = 0.6, n = 500$; (j): $\lambda = 0.6, n = 1000$;

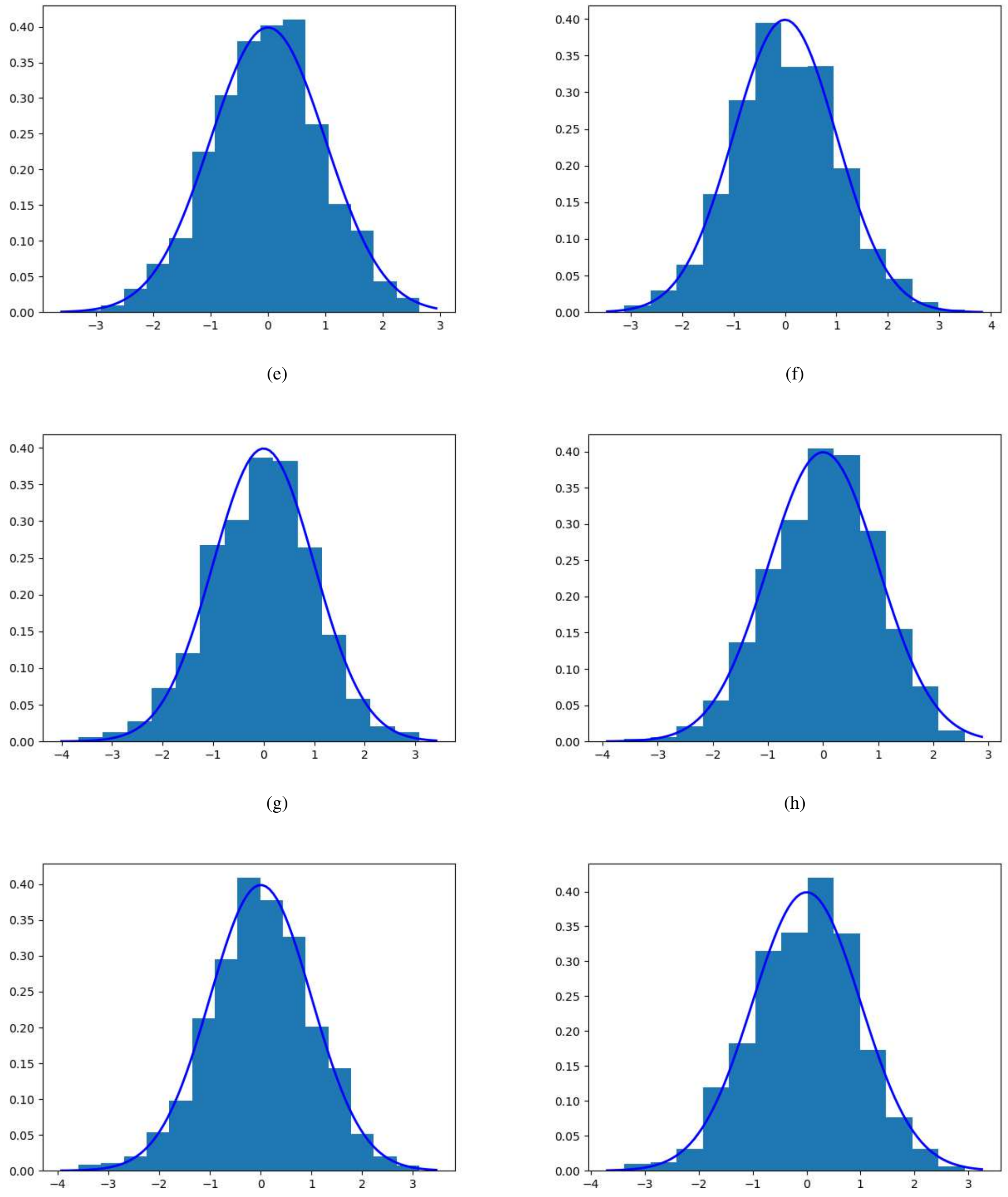

(e) (f)

(g) (h)

(i) (j)
